# Macular OCT’s Proficiency in Identifying Retrochiasmal Visual Pathway Lesions in Multiple Sclerosis—A Pilot Study

**DOI:** 10.3390/diagnostics14121221

**Published:** 2024-06-09

**Authors:** Larisa Cujbă, Ana Banc, Cristina Stan, Tudor Drugan, Cristina Nicula

**Affiliations:** 1 Medical Doctoral School, University of Oradea, 410087 Oradea, Romania; larisacujba@gmail.com; 2 Department of Ophthalmology, “Iuliu Hatieganu” University of Medicine and Pharmacy, 400006 Cluj-Napoca, Romania; 3 Department of Medical Informatics and Biostatistics, “Iuliu Hațieganu” University of Medicine and Pharmacy, 400349 Cluj-Napoca, Romania; 4 Department of Maxillo-Facial Surgery and Radiology, “Iuliu Hațieganu” University of Medicine and Pharmacy, 400012 Cluj-Napoca, Romania; niculacristina65@yahoo.com

**Keywords:** optical coherence tomography, neurodegeneration, macular ganglion cell layer, inner plexiform layer, multiple sclerosis, retrochiasmal lesion, visual pathway

## Abstract

Optical coherence tomography (OCT) is a non-invasive imaging technique based on the principle of low-coherence interferometry that captures detailed images of ocular structures. Multiple sclerosis (MS) is a neurodegenerative disease that can lead to damage of the optic nerve and retina, which can be depicted by OCT. The purpose of this pilot study is to determine whether macular OCT can be used as a biomarker in the detection of retrochiasmal lesions of the visual pathway in MS patients. We conducted a prospective study in which we included 52 MS patients and 27 healthy controls. All participants underwent brain MRI, visual field testing, and OCT evaluation of the thicknesses of the peripapillary retinal nerve fiber layer (pRNFL), macular ganglion cell layer (GCL), and macular inner plexiform layer (IPL). OCT measurements were adjusted for optic neuritis (ON). VF demonstrated poor capability to depict a retrochiasmal lesion identified by brain MRI (PPV 0.50). In conclusion, the OCT analysis of the macula appears to excel in identifying retrochiasmal MS lesions compared to VF changes. The alterations in the GCL and IPL demonstrate the most accurate detection of retrochiasmal visual pathway changes in MS patients.

## 1. Introduction

Multiple sclerosis (MS), the most common chronic inflammatory and immune-mediated disorder of the central nervous system (CNS), is a challenging condition in terms of diagnosis and monitoring [1,2]. The triad comprising demyelination, axonal loss, and neurodegenerative processes represents a constant characteristic of MS [2,3].

In recent years, optical coherence tomography (OCT) has emerged as a non-invasive and cost-effective imaging technique, shedding new light on the intricate connections between ocular structures and MS-related pathology [4,5].

OCT, renowned for its precision and reproducibility, has the ability to discern microstructural features of neural tissue, specifically within the optic nerve head and retina, thus positioning OCT as a vital tool for the investigation of neurodegenerative processes [6]. Its utility was first documented in 1999, when peripapillary retinal nerve fiber layer (pRNFL) thinning was observed in MS patients with a history of optic neuritis (ON) [7].

### 1.1. The Impact of MS on the Visual Pathway

MS demyelinating lesions have the potential to impact the afferent visual pathway extensively, spanning from the optic nerve to the primary visual cortex [8].

While approximately 25% of patients with clinically isolated syndrome (CIS) exhibit ON as the first clinical manifestation (it may occur during the course of the disease in 75% of patients with MS), MS frequently affects the posterior visual pathway with the involvement of optic radiations due to their location in the periventricular white matter, a common site for MS lesions [9,10].

#### 1.1.1. Anatomy of the Visual Pathway

The starting point of the anterior visual system lies in the retina. The long axons of the retinal ganglion cells (RGCs) constitute the optic nerve, optic chiasm, and optic tract (OT). Converging on the lateral geniculate nucleus (LGN), where synaptic connections are made, the pathway continues with the axons of the next-level neurons that form optic radiations, which project onto the visual cortex. The retrogeniculate visual pathway is also known as the posterior visual pathway [8,11].

The unmyelinated nature of the retina and the axons in the innermost retinal layer provide a unique opportunity to study neurodegeneration within the CNS [12].

#### 1.1.2. Trans-Synaptic Degeneration

Neurodegeneration due to the lesions of the visual pathway is propagated either retrograde (i.e., toward the retina) or anterograde (i.e., toward the occipital cortex). Trans-synaptic degeneration has also been described, with neurodegeneration being transmitted past the synaptic level within the LGN [11]. Understanding this process is crucial to comprehending the impact of MS lesions on the visual pathway.

### 1.2. Importance of Perimetry in MS

The assessment of the visual pathway’s integrity involves various tests, such as high-contrast visual acuity, low-contrast visual acuity (LCVA), contrast sensitivity, color vision acuity, and visual field (VF) [13].

The afferent visual pathway, meticulously organized from RGC bodies to the occipital cortex, exhibits predictable VF defects (VFDs) when lesions occur in specific anatomic locations. OCT detects structural changes in the optic nerve often preceding VF alterations, emphasizing the importance of performing and interpreting VF and OCT results together for precise lesion localization [14].

A significant finding from research conducted by Vidovic et al. in 2005 was the presence of VFDs in MS patients without prior history of ON [15]. These defects, situated in the peripheral regions of the VF, demonstrated a gradual progression, often going unnoticed by the affected individuals. This emphasizes the critical role of perimetry in the early identification of VFDs in MS patients, offering a proactive approach to their detection even before subjective disturbances manifest [15].

The patterns of VFDs provide important clues for the precise localization of the lesions of the afferent visual pathway. Unilateral prechiasmal lesions typically manifest as nerve fiber bundle-type patterns, whereas chiasmal and retrochiasmal disorders commonly result in bilateral VFDs with respect to the vertical meridian [14,16]. Understanding these patterns is essential to revealing the underlying disorder and adjusting interventions accordingly.

### 1.3. Optical Coherence Tomography in MS

#### 1.3.1. OCT as a Gateway to Neurological Assessment

The exact anatomical configuration of the retina, coupled with the sustained preservation of retinoscopic organization along the afferent visual pathway, enhances the utility of OCT in evaluating CNS diseases [17]. This detailed assessment involves both the optic nerve and the retina due to their shared embryologic origin within the CNS [18].

#### 1.3.2. Global Retinal Changes in MS

A noteworthy finding is that almost every individual with MS will exhibit a decline in pRNFL and thinning of the macular ganglion cell layer (GCL) over time, irrespective of ON history [5]. Recent research suggests synaptic injury preceding neuronal loss in MS, with early thinning of the inner plexiform layer (IPL) identified as a potential independent biomarker for disease progression [19].

Additionally, the inner nuclear layer (INL) is of particular interest for examining inflammation in MS pathophysiology [20]. Recently, the INL thickness has been identified as a potential marker for inflammatory activity in both relapsing–remitting MS (RRMS) and progressive MS patients exhibiting MRI activity [21,22]. Microcystic macular edema, affecting 4.7% of individuals with MS, involves the INL of the retina and is linked to disease severity [23]. However, research findings are contradictory, with some studies reporting INL thinning and others reporting thickening in MS patients [21,23,24,25,26]. However, early signs of inflammation may manifest in the INL before progressing to subsequent neurodegeneration [27].

#### 1.3.3. Macular Ganglion Cell and pRNFL Loss beyond Optic Neuritis

Advanced OCT segmentation software enhances understanding of axonal degeneration in MS by objectively examining all retinal layers. Recent research indicates that the GCL is a key biomarker for early diagnosis, severity, and progression monitoring [28].

Assessment of the pRNFL and macular GCL and IPL holds great promise for enhancing MS diagnosis, severity assessment, and progression monitoring. These measurements aid in disability-worsening prediction and reveal accelerated retinal atrophy in younger MS patients, supporting OCT’s role in documenting neuroprotection outcomes and guiding therapeutic strategies [29,30,31,32]. Moreover, they underscore its clinical relevance in detecting remyelination and understanding disease pathology [33].

While ON is traditionally recognized as a primary contributor to GCL, IPL, and pRNFL loss in MS, recent findings have unveiled such losses in eyes without ON [20]. The multifaceted etiology encompasses factors like subclinical optic nerve inflammation, primary retinal degeneration, or trans-synaptic transmission of damage from the posterior visual pathway [10,34]. Some papers investigated a direct autoimmune mechanism associated with retinal antigens, suggesting a potential fundamental pathophysiological basis for pRNFL thinning in MS [35].

The ability of OCT to measure the retina and optic nerve in vivo, combined with structure–function correlations in the afferent visual system of MS patients, makes the OCT analysis of the retinal structure a useful model for tracking and assessing neurodegeneration.

In the diagnostic assessment of retrochiasmal lesions, relying solely on pRNFL examination proved insufficient. While pRNFL assessment remains valuable, the detection of GCL atrophy is of paramount importance. The hemi-macular atrophy of GCL is a crucial marker of retrograde trans-synaptic degeneration [36].

### 1.4. Research Objectives

The purpose of this pilot study is to determine whether macular OCT can be used as a biomarker in the detection of retrochiasmal lesions of the visual pathway in MS patients.

## 2. Materials and Methods

### 2.1. Study Design

A prospective, observational, and monocentric study was conducted at the Departments of Ophthalmology and Neurology, Cluj County Emergency Clinical Hospital (Cluj-Napoca, Romania). The selection of the participants was based on convenience sampling, with the data collection performed prospectively. The study protocol was approved by the Institutional Review Board, and the research adhered to the Declaration of Helsinki. Written informed consent was obtained from all participants prior to their enrollment in the study.

### 2.2. Participants

We included patients with either a confirmed diagnosis of clinically definite RRMS or CIS according to the 2017 McDonald criteria [37], as well as a group of healthy age- and sex-matched controls.

Exclusion criteria comprised (1) patients with overlapping ocular or neurological disorders, such as dense ocular media opacities, macular disease, history of posterior pole surgery, cranio-cerebral trauma, stroke, meningitis, encephalitis, or brain tumors; (2) patients with poor-quality SD-OCT images (e.g., motion artifacts, excessive blinking); and (3) patients unable to provide written informed consent.

### 2.3. Clinical and Paraclinical Assessment

We collected the following demographic data: age, gender, clinical form of MS, time since diagnosis, and history of ON.

Each participant underwent a standard ophthalmic examination, including best-corrected visual acuity measured using the Snellen chart, slit-lamp evaluation of the anterior and posterior pole, Goldmann applanation tonometry, computerized perimetry, and OCT examination. Data were collected from both eyes of each participant.

Additionally, all RRMS and CIS patients underwent contrast-enhanced brain magnetic resonance imaging (MRI) within 3 months prior to study enrollment.

#### 2.3.1. OCT Measurement Methodology

OCT measurements were conducted in both eyes of each participant by the same technician utilizing the Spectral Domain OCT device (Spectralis, Heidelberg Engineering GmbH, Heidelberg, Germany) after pupil dilation with 1% tropicamide. This device is equipped with an integrated real-time eye-tracking system, which monitors eye movements and stabilizes the scan position.

Scans of both the optic nerve and macular regions were captured by employing the predefined ONH-RC and PPoleH protocols, respectively (ONH-RC, optic nerve head—radial and circle scans; PPoleH, posterior pole horizontal scans).

The Spectralis OCT device performs an automated segmentation of the retinal layers, which was meticulously inspected for segmentation errors and manually refined if needed by the same experienced operator.

The pRNFL analysis involved scanning a 3.5 mm-diameter circumferential ring centered on the optic nerve head, generating a chart divided into 6 sectors (TS—temporal superior, T—temporal, TI—temporal inferior, NI—nasal inferior, N—nasal, and NS—nasal superior) [26]. Each quadrant presented the mean thickness value of the pRNFL in microns. The average pRNFL thickness was also provided.

**Figure 1:**
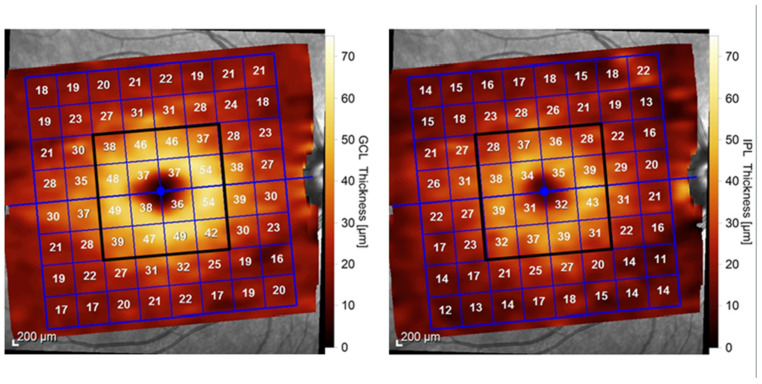
SD-OCT macular scan using posterior pole analysis of the right eye of an MS patient: a 8 × 8 grid (blue square) of sectors displaying numerical values in microns, representing the thickness of each sector in the GCL. We included in the analysis the 16 sectors corresponding to the central 4 × 4 grid (black square) of the GCL (**left**) and IPL (**right**). SD-OCT, spectral domain optical coherence tomography; GCL, ganglion cell layer; IPL, inner plexiform layer.

The PPoleH protocol involved 61 horizontal B-scans generating full-layer retinal thickness maps and segmented thickness maps displayed in 8 × 8 grid mode [38]. Within the 8 × 8 grid, we assessed and averaged the retinal thickness in the central 24° area of the posterior pole. We only included the central 4 × 4 grid in the analysis, corresponding to GCL and IPL thicknesses (Figure 1). The 16 sectors were labeled and numbered for each layer according to their divisions into temporal, nasal, superior, and inferior regions relative to the fovea.

In this study, we exclusively examined the thicknesses of the retinal layers known to correlate with neurodegeneration in MS, namely, the GCL and the IPL, as demonstrated in previous research [20,39]. We recorded the thicknesses of the GCL and IPL in each of the 16 central macular sectors, as provided by the Spectralis device. The thickness values were expressed in micrometers, and each layer’s thickness map was used for the interpretation of the results.

#### 2.3.2. Visual Field Testing

VF was assessed for each eye separately using the 30-2 SITA Fast test of the Humphrey Field Analyzer (Carl Zeiss Meditec, Dublin, CA, USA) with appropriate refractive correction using trial lenses mounted in the equipment-incorporated lens holder.

All participants were offered a training session. Reliable VFs were considered those with less than 33% fixation losses and false-positive and false-negative responses.

The clinical interpretation of the VF of every MS patient aimed to evaluate the presence of VF loss. Results were categorized as either yes or no, indicating the presence or absence of visual alterations in each eye separately.

#### 2.3.3. Neuroimaging

Brain MRI investigations were conducted on all patients within three months prior to study enrollment to evaluate the extent and nature of the CNS involvement. The imaging protocol included T1-weighted, T2-weighted, and fluid-attenuated inversion recovery (FLAIR) sequences to assess lesion burden and brain atrophy. T1-weighted and T2-weighted images were obtained in the axial plane, while FLAIR sequences were performed axially and sagittally. Additionally, contrast-enhanced T1-weighted images were captured to identify active lesions. All images were analyzed for the presence, number, and volume of lesions in various brain regions, including periventricular, juxtacortical, and infratentorial areas, as well as for any signs of brain atrophy. All scans were reviewed by experienced neuroradiologists to ensure accurate interpretation. All MRI images were then analyzed by the same investigator, who was masked to the OCT and VF results. The analysis of MRI images involved documentation of the presence of lesions along the retrochiasmal visual pathways and whether they predominantly affected the right or left optic pathway.

### 2.4. Statistical Analysis

As ON is often found in MS patients’ history, we adjusted the values of the macular OCT parameters in this category of patients. We calculated the mean difference of macular OCT values between patients with a history of ON and controls, and the obtained difference was added to the macular OCT values of patients with a history of ON. We performed the adjustment for each sector of the GCL and IPL 4 × 4 grids.

After we completed the corrections for ON, we conducted a patient-level OCT analysis by evaluating the GCL and IPL thickness maps of both eyes. The OCT results were interpreted as indicative of a retrochiasmal lesion of the visual pathways if GCL or IPL alterations were present in the hemi-maculae of the same side—i.e., GCL or IPL loss present in the temporal (or right) hemi-macula of the right eye and in the nasal (or right) hemi-macula of the left eye, or vice versa (left hemi-maculae in both eyes). This analysis was performed by two independent ophthalmologists who were masked to the VF and brain MRI results; the OCT interpretations were discussed afterwards to ensure consensus for each patient.

In order to validate the contingency tables used to obtain the sensitivity and specificity of macular OCT and brain MRI as diagnostic tests for retrochiasmal MS lesions, we used the McNemar test. Additionally, we used the McNemar test in the same way for the contingency tables for VF and brain MRI, and between macular OCT and VF.

Lastly, we performed an eye-level analysis in which we intersected macular OCT with brain MRI diagnosis by considering whether the eye was ipsilateral or contralateral to the MRI lesion.

## 3. Results

We included 52 patients and 27 healthy controls. The demographic data of all participants are included in Table 1.

**Figure 2:**
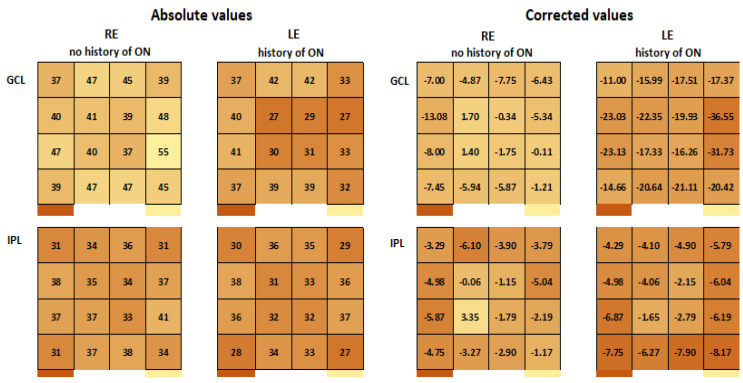
Mean thicknesses of GCL and IPL sectors before and after correction. Each sector was color-coded depending on the thickness values—the darker the color, the thinner the retinal layer. On the (**left**) side of the image, the absolute numerical thickness values for each sector of the 4 × 4 macular grid for the two retinal layers of interest, GCL and IPL, are recorded, as examined using posterior pole analysis. These values are provided for both the RE and the LE of the same patient, with the LE having previously experienced an episode of ON. On the (**right**) side of the image, the corrected numerical thickness values for each sector of the 4 × 4 macular grid for the two retinal layers of interest, GCL and IPL, are displayed. These values underwent mathematical corrections: One correction was applied to the right eye, involving the subtraction of the mean thickness value of the controls (as this eye has no history of optic neuritis), while two corrections were applied to the LE. These involved both subtracting the mean thickness value of the controls and adding the mean calculated thinning caused by ON in the LE. The IPL seems to be more sensitive to MS damage. RE, right eye; LE, left eye; GCL, ganglion cell layer; IPL, inner plexiform layer; ON, optic neuritis.

In Figure 2 we present the mean thicknesses for each analyzed sector of GCL and IPL of all patients before and after correction. Each sector was color-coded depending on the thickness values—the darker the color, the thinner the retinal layer. Similar graphic representations were obtained for each eye of each patient and were used for the interpretation of OCT results as described in the previous section.

Our correction method comprised two steps:

- Baseline adjustment: We subtracted the average layer thickness of the control group (healthy subjects) from each sector in all groups. This aimed to center all thickness values around zero for easier comparison.
- ON adjustment (for ON patients only): We introduced an additional adjustment specific to ON patients. We added the average difference in layer thickness between ON patients and non-ON patients for each sector. This step compensated for the thickness changes specifically caused by the presence of ON, allowing us to focus on other variations in layer thickness.

The results of the patient-level comparison between macular OCT and brain MRI are reported in Table 2. The results obtained for the positive predictive value (PPV) show a good capability of macular OCT GCL or IPL to depict a retrochiasmal lesion identified by brain MRI, with greater values obtained using the OCT IPL values corrected for ON. The *p*-value was less than 0.001, indicating that there is a statistically significant difference between the OCT and MRI methods in terms of their discordant pairs.

The results of the at patient-level comparison between VF and brain MRI are reported in Table 3. The results show a poor capability of VF to depict retrochiasmal lesions identified by brain MRI.

In Table 4, we report the results of the patient-level comparison between macular OCT and VF. The results obtained for the PPV are weak and are due to the poor capability of VF to identify retrochiasmal lesions shown on brain MRI. The *p*-value was greater than 0.05, which indicates that there is no statistically significant difference between macular OCT and VF methods in terms of their discordant pairs.

The results of the eye-level comparison between macular OCT and brain MRI are reported in Table 5. The high values obtained for the PPV show that macular OCT has good capability to depict retrochiasmal lesions identified by brain MRI.

## 4. Discussion

The investigation of the relationship between OCT, VF, and brain MRI in patients with MS is an important research topic. Petzold et al. [40] suggested that asymptomatic lesions of the visual pathway are a major cause of retinal asymmetry in MS patients. Many MS-associated lesions are asymptomatic, underscoring the significance of early detection [41]. The cost and accessibility issues of MRI for MS monitoring, along with difficulties in distinguishing demyelination from neurodegeneration, emphasize the necessity of alternative diagnostic tools. OCT shows potential as a cost-effective tool for the evaluation of neurodegenerative disorders [42,43].

The most recent revision of MS diagnostic guidelines focuses on the investigation of the diagnostic capabilities of retinal OCT [37]. Two meta-analyses provide evidence for the consistency of OCT measurements associated with retinal-layer degeneration. This evidence strongly supports the integration of OCT into the diagnostic assessment of MS patients [20,44].

### 4.1. OCT in the Evaluation of MS Patients

Retinal thinning is mainly linked to damage in the optic nerve and can be seen in different clinical MS phenotypes [45,46,47]. Less severe inner retinal atrophy is associated with damage in the retinal–cortical connections, with the periventricular white matter often being a site affected by MS lesions [41,48]. Notably, MS-related ON is identified as the primary factor behind the asymmetric atrophy observed in the inner retinal layers [40].

The measurement of GCL thickness in the macular area, where 40% of RGCs are found, is a sensitive parameter for assessing diseases related to GC damage. This approach is more sensitive in detecting sectoral neuronal loss compared to pRNFL measurements due to the complex distribution of nerve fibers around the optic disc. The lack of specificity between VF loss and optic disc sectors, especially in patients with small quadrant lesions, underscores the superior sensitivity of GCL measurements in this context [49].

Post-geniculate lesions may result in homonymous macular GCL and IPL thinning due to trans-synaptic retrograde degeneration. It is important to note that this particular OCT pattern changes gradually and may take over a year to become noticeable [50]. This pattern of homonymous atrophy of GCL and IPL can be observed in OT, LGN, and retrogeniculate lesions [48,51].

GCL and IPL thinning may precede the thinning of the pRNFL and, in some cases, precede changes detected by standard automated perimetry (SAP) [50,52,53].

According to the IMSVISUAL study (2018), people with a pRNFL thickness of ≤87 or ≤88 μm (measured with Cirrus HD-OCT and Spectralis OCT) have double the risk of disability worsening in the first three years and almost quadruple the risk from year three to year five. Significantly, there were no important connections found for macular volume, and the research did not have information on GCL or IPL thickness to be included in the study [54]. Recently, Cordano et al. reported that synaptic injury within the IPL precedes disease progression from relapsing–remitting MS to secondary progressive MS, correlating with IPL thinning observed in OCT scans [19].

Our study supports the existing literature by highlighting the value of macular OCT as a marker for identifying retrochiasmal lesions in MS patients [55,56,57,58]. By measuring the thickness of the GCL and IPL, macular OCT shows strong potential for detecting retrochiasmal MS lesions, as shown by the PPV analysis. Moreover, the statistically significant difference between the OCT and MRI methods underscores the potential of macular OCT as a complementary tool in MS diagnosis and monitoring. These findings suggest that incorporating macular OCT into clinical practice could improve the identification of retrochiasmal lesions in MS patients and the management of disease progression.

Moreover, our findings could reveal an intriguing aspect of MS pathology: The retinal changes observed in the eye opposite to the retrochiasmal lesion are more prominent than those in the ipsilateral eye. This phenomenon could be explained by the disproportionate distribution of crossed and uncrossed fibers in the optic chiasm, with approximately 53% crossed and 47% uncrossed fibers [59]. Consequently, when a retrochiasmal lesion occurs, it disrupts both types of fibers. However, due to the higher proportion of crossed fibers, the impact on the contralateral eye is more significant.

Since our results showed a high PPV against a low sensitivity for the diagnosis value of OCT compared with MRI, we can expect that the use of OCT will result in a certain level of non-diagnosed patients (false negatives).

Some recent papers introduced voxel-based morphometry (VBM) as an advanced imaging tool that improves OCT’s capability to detect retinal changes in MS [60]. VBM is an imaging analysis technique that allows for investigation of focal differences in the studied tissue by segmentation into voxels (i.e., 3D pixels) [60]. VBM-OCT reliably identifies atrophy in the pRNFL and GCL, especially in the nasal macula, even in early MS stages. The specific patterns of pRNFL and GCL thinning suggest retrograde degeneration from RGC axons to their cell bodies. This nuanced insight underscores the value of topographical information in assessing early neurodegeneration in MS, thereby increasing OCT’s sensitivity in detecting subtle changes linked to disease progression [60].

### 4.2. VF Assessment in MS Patients

Our study underscores the limitations of VF testing in the detection of retrochiasmal lesions in MS patients compared to brain MRI findings. The weak PPV obtained for VF indicates its poor capability to accurately depict retrochiasmal lesions observed in MRI. Moreover, the lack of a statistically significant difference between macular OCT and VF methods in terms of their discordant pairs suggests that VF does not outperform macular OCT in this context. Our results emphasize the limitations of the VF in the detection of retrochiasmal lesions and highlight the importance of complementary imaging techniques such as macular OCT to improve the diagnostic accuracy and management of MS-related visual impairment.

Perimetry in neuro-ophthalmology serves various purposes, including diagnosis, monitoring, and functional assessment [61]. Commonly reported programs, such as the 30–2 and 24–2 programs of the Humphrey perimeter, alongside manual kinetic perimetry using the Goldmann perimeter, are essential for assessing VFs in neurological conditions [62].

Ortiz-Perez et al.’s study reported strong connections between pRNFL and GCL thicknesses, as well as VF mean deviation [63]. Even though individuals with abnormal VF test results had thinner retinal layers compared to those with normal VF results, the difference was not statistically significant [63]. This study highlights the association between initial VF deficiency and accelerated disability advancement in MS during a 3-year monitoring period, highlighting the importance of VF assessment in the evaluation of CNS deterioration in MS [63]. Chorazy et al. (2007) emphasize the common occurrence of asymptomatic VF disturbances in MS patients without ON history, showing associations with disease duration and disability levels [64]. This underscores the importance of continuous visual monitoring in MS, even in the absence of apparent ON.

Previous reports have shown associations between retinal imaging and LCVA and color vision scores in non-ON eyes of MS patients [65,66]. However, these associations were absent in non-ON eyes using standard white-on-white perimetry [67,68]. This suggests that VF abnormalities may originate from damage along the entire visual pathway, while RGC damage predominantly contributes to LCVA abnormalities and dyschromatopsia [69,70].

Correlations between mean deviation of VF, brain parenchymal volume, and retinal thickness imply neuroaxonal damage as a significant histopathological factor in VF impairment. Additionally, diffuse demyelination, a significant factor in CNS inflammation in MS, can lead to dysfunction and maldistribution of ion channels, resulting in excitotoxicity, mitochondrial dysfunction, and energy failure, and ultimately resulting in neuroaxonal damage [71].

Another study [72] emphasized that OCT unveils more extensive RGC axonal damage than reported functional deficits in ON-MS patients. This prompts a reconsideration of the advantages of structural tests like OCT over functional tests such as SAP for optic nerve damage assessment. Although SAP and OCT were consistent in identifying ON eyes, their agreement was weaker for non-ON eyes and specific areas. Disagreement arises from factors like test sensitivity, specificity, and inherent limitations. Additionally, the study highlights the limitation of SAP measuring the entire visual pathways, while OCT focuses on RGC axonal integrity. This is crucial, as MS may involve central visual pathways not leading to retrograde degeneration of the pRNFL, thus contributing to the observed discrepancies. Effective disease monitoring and treatment evaluation relies on a thorough understanding of the structure–function relationship [72].

While symptomatic retrochiasmal lesions leading to homonymous VFDs are rare (1.3–3.5%), MRI reveals frequent retrochiasmal demyelinating lesions (30–90%) [73,74]. It is worth mentioning that larger lesions are predictive of homonymous VFDs, suggesting potential underdiagnosis.

In MS patients presenting with homonymous VFDs, the majority are young individuals with RRMS. Partial homonymous VFDs are more common than complete homonymous VFDs, influenced by the size and location of demyelinating lesions. Lesions in the optic radiations, often small and asymptomatic, contribute to partial homonymous VFD prevalence. Compact OT axons are more likely to cause complete homonymous VFDs, while the dispersed axons in the optic radiations often lead to partial HVFDs in MS. The majority of lesions associated with homonymous VFDs are small and found in the optic radiations, explaining why partial homonymous VFDs are more common. MRI studies have shown a greater occurrence of retrochiasmal demyelinating lesions compared to homonymous VFDs, which suggests that optic radiations lesions can often be asymptomatic [74].

The difference between clinical and anatomical data suggests potential underdiagnosis of homonymous VFDs in MS patients. Schmutz and Borruat (2020) highlight the high occurrence of partial homonymous VFDs due to small asymptomatic lesions in the optic radiations [74]. While most cases of homonymous VFDs typically fully recover within ten weeks, around 23% of individuals experience only partial recovery after an average of three months. The progression of RRMS to a secondary progressive phase is suggested by the worsening of a homonymous VFD in the absence of a new relapse [74].

### 4.3. Limitations

In discussing the limitations inherent to our study, several factors warrant consideration. Firstly, the inclusion of patients with ON poses a challenge in the interpretation of the retinal thickness measurements, as the need to compensate for optic nerve damage may influence these metrics. Despite efforts to account for ON damage, its impact on retinal thickness remains a potential confounding factor. Additionally, it is important to recognize the limitations of ocular OCT in depicting MS lesions that do not directly involve the visual pathway, which may restrict the generalization of our results. Moreover, our decision to focus solely on the central 4 × 4 macular grid rather than the full 8 × 8 grid for macular analysis may limit our ability to detect other potential abnormalities within the retinal structure. The exclusion of pRNFL analysis due to the arrangement of retinal fibers in the optic nerve head represents another constraint. Lastly, another limitation of our study is that it did not include data related to color vision.

While acknowledging these limitations, our study provides valuable insights into retinal changes associated with MS, highlighting areas for future research to refine our understanding and improve clinical management strategies.

## 5. Conclusions

The OCT analysis of the macula appears to excel in identifying retrochiasmal MS lesions compared to VF changes. The alterations in GCL and IPL demonstrate the most accurate detection of retrochiasmal visual pathway changes in MS patients.

## Figures and Tables

**Table 1:** Demographic data of the participants included in the study.

Variable	Patients n = 52	Controls n = 27
Age (years), mean ± SD	35.07 ± 12.73	31.85 ± 10.97
Gender, men n (%)	12 (23%)	6 (22.2%)
Clinical form of MS, n (%) • RRMS • CIS	45 (86.54%) 7 (13.46%)	0 0
Time since MS diagnosis (years), mean ± SD	4.3 ± 5.39	0
History of ON, n (%)	16 (30.7%)	0

**Table 2:** Patient-level comparison between macular OCT and brain MRI as diagnostic methods for retrochiasmal MS lesions.

Comparison	Sensitivity	Specificity	Positive Predictive Value	Negative Predictive Value	McNemar Test *p*-Value
GGL vs. MRI	0.39	0.68	0.78	0.27	<0.0001
Corrected GGL vs. MRI	0.40	0.63	0.76	0.26	<0.0001
IPL vs. MRI	0.45	0.68	0.80	0.30	<0.0001
Corrected IPL vs. MRI	0.48	0.68	0.81	0.31	0.0001

**Table 3:** Patient-level comparison between VF and brain MRI as diagnostic methods for retrochiasmal MS lesions.

Comparison	Sensitivity	Specificity	Positive Predictive Value	Negative Predictive Value	McNemar Test *p*-Value
VF vs. MRI	0.14	0.62	0.50	0.22	<0.0001

**Table 4:** Patient-level comparison between macular OCT and VF as diagnostic methods for retrochiasmal MS lesions.

Comparison	Sensitivity	Specificity	Positive Predictive Value	Negative Predictive Value	McNemar Test *p*-Value
GGL vs. VF	0.33	0.73	0.25	0.80	0.36
Corrected GGL vs. VF	0.38	0.74	0.29	0.81	0.34
IPL vs. VF	0.33	0.77	0.28	0.81	0.70
Corrected IPL vs. VF	0.38	0.77	0.31	0.82	0.55

**Table 5:** Eye-level comparison between macular OCT and brain MRI as diagnostic methods for retrochiasmal MS lesions.

Comparison	Sensitivity	Specificity	Positive Predictive Value	Negative Predictive Value	McNemar Test *p*-Value
GGL vs. MRI (ipsilateral lesion)	0.39	0.66	0.68	0.37	0.0005
Corrected GGL vs. MRI (ipsilateral lesion)	0.39	0.60	0.64	0.34	0.002
IPL vs. MRI (ipsilateral lesion)	0.48	0.70	0.75	0.42	0.002
Corrected IPL vs. MRI (ipsilateral lesion)	0.50	0.66	0.73	0.41	0.005
GGL vs. MRI (contralateral lesion)	0.41	0.73	0.78	0.35	<0.0001
Corrected GGL vs. MRI (contralateral lesion)	0.43	0.69	0.76	0.34	0.0001
IPL vs. MRI (contralateral lesion)	0.46	0.69	0.77	0.36	0.0003
Corrected IPL vs. MRI (contralateral lesion)	0.50	0.69	0.78	0.37	0.0007

## Data Availability

Data are available on request from the authors.
